# The Cigarette Evil

**Published:** 1892-11

**Authors:** 


### THE CIGARETTE EVIL.

Considering what very poor things cigarettes are, it is surprising that they should have got such a hold on the community. But, bad as they are, they are extremely fascinating. The use of them, when carried to excess, becomes a habit that is most difficult to break, while they are so cheap and so convenient that it takes exceptional discretion to smoke them at all without smoking them to a deleterious extent. Of course it is primarily because they are so cheap that they appeal so generally to boys ; but even with boys, who ought not to be allowed to smoke at all, it is not so much the tobacco in the cigarette that does the mischief as the pestilent and insinuating practice of inhaling the smoke. An ordinary boy of wholesome appetites won't smoke cigars or pipe tobacco enough to do him serious damage, even if he can get them, nor would the cigarettes he might smoke be so serious a menace to his welfare if he could only smoke them as he would smoke cigars. The trouble is that as soon as he gets used to cigarette smoking he begins to inhale the smoke, and presently is fixed in a habit that plays the mischief with him.

Whether any thing besides tobacco goes into ordinary cigarettes is a much discussed question. The effect they sometimes produce on the brain is so different from that due to tobacco in other forms as to favor the theory that many of them contain opium or valerian ; but this the manufacturers deny, usually asserting that such drugs are too expensive to put into cheap cigarettes, even if it helped their marketable qualities. One thing besides the tobacco obviously goes into them, and that is the paper, the fumes of which are, doubtless, bad for the throat and lungs as far as they go.

